# Accounting for the increase in NSAID expenditure: substitution or leakage?

**DOI:** 10.1186/1478-7547-4-9

**Published:** 2006-05-31

**Authors:** Garry R Barton, Anthony J Avery, David K Whynes

**Affiliations:** 1 School of Economics, University of Nottingham, Nottingham, NG7 2RD, UK; 2 Division of Primary Care, Medical School, University of Nottingham, Queen's Medical Centre, Nottingham, NG7 2UH, UK

## Abstract

**Background:**

National Institute of Health and Clinical Excellence (NICE) guidance stated that a new form of non-steroidal anti-inflammatory drug (NSAID) (selective COX-2 inhibitors) should only be an option for arthritis patients at high risk of a gastro-intestinal (GI) event. Total expenditure on NSAIDs has risen by 57% over five years, to £247 million in 2004. We assess whether this expenditure increase can be accounted for by substitution – an increased prescribing of two (more expensive) selective COX-2 inhibitors (celecoxib and rofecoxib) and a simultaneous equivalent reduction in the prescribing volume of three (cheaper) older NSAIDs (diclofenac, ibuprofen and naproxen).

**Methods:**

Quarterly prescription data was collated from January 1999 to September 2004. Over this period, the level of correlation between the total prescribing volumes for i) celecoxib and rofecoxib, and ii) diclofenac, ibuprofen, and naproxen were compared, the change in total expenditure on the five NSAIDs was also estimated. The latter was apportioned into that which was estimated to have arisen due to i) substitution, and ii) increased NSAID prescription volume.

**Results:**

Total prescription volumes for the two NSAID groups were negatively correlated (r = -0.97, p < 0.001). In the last quarter there were 1.23 million prescriptions for celecoxib and rofecoxib, and 0.46 million fewer prescriptions for naproxen, diclofenac, and ibuprofen (than in the first quarter, when celecoxib and rofecoxib were not prescribed). Total expenditure for the five NSAIDs was £32.7 million higher in the last quarter, than the first, £12.2 million of which was estimated to be due to substitution, and £20.4 million due to increased volume.

**Conclusion:**

The introduction of celecoxib and rofecoxib was associated with a reduction in the prescription volume for naproxen, diclofenac, and ibuprofen. However, overall quarterly prescription volume for these five NSAIDs increased by 0.76 million, and we estimate that quarterly expenditure increased by £20.4 million more than would have been expected if overall NSAID volume had remained constant. This suggests that the prescription of both celecoxib and rofecoxib may have 'leaked' to population groups who would not previously have received an older NSAID.

## Background

Non-steroidal anti-inflammatory drugs (NSAIDs) are used extensively in the health service to relieve conditions that have an inflammatory component, and to relieve pain. In England, over 20 million NSAID treatments were prescribed in 2004, and NSAID expenditure amounted to £247 million (3.1% of annual prescription expenditure) [1].

NSAIDs can however induce adverse events – in the United Kingdom (UK) it has been estimated that each year they cause 3,500 adverse gastro-intestinal (GI) events (perforations, ulcers, bleeds, etc.) which require hospitalisation, and 400 deaths [2]. A gastro-protective agent (GPA) can be co-prescribed in an attempt to reduce the risk of a GI event, however, more recently, a new form of NSAID (the selective cyclooxygenase-2 (COX-2) inhibitor) has been developed. Selective COX-2 inhibitors aim to inhibit the COX-2 enzyme (which is responsible for inflammation), without inhibiting the COX-1 enzyme (which helps to protect the mucosa lining of the stomach and other parts of the gastro-intestinal tract) [3]. In contrast, older NSAIDs inhibit both the COX-2 and COX-1 enzymes, thus creating increased risk of adverse GI events.

In 2000, the National Institute of Clinical and Health Excellence (NICE) undertook an assessment of the available evidence on the health benefits and costs of selective COX-2 inhibitors for arthritis patients [4]. Though the assessment found no evidence that any of the (four) COX-2 inhibitors were clinically superior to one another, it did conclude that selective COX-2 inhibitors had equivalent efficacy to NSAIDs (in terms of their ability to reduce pain and improve physical functioning) and that selective COX-2 inhibitors were associated with fewer GI events than other NSAIDs [4]. On the basis of this evidence, NICE recommended that selective COX-2 inhibitors should not be routinely used (in preference to an older NSAID) by patients with arthritis, but that they should be an option for those who are at high risk of a GI event [5]. By December 2004 annual expenditure on selective COX-2 inhibitors had grown to over £150 million in England, and overall NSAID expenditure was approximately £65 million higher than in 2001 [1].

In this paper we seek to determine whether the aforementioned increase in overall NSAID expenditure can be accounted for by substitution i.e. the increased prescribing of (more expensive) selective COX-2 inhibitors and a simultaneous equivalent reduction in the prescribing volume of (cheaper) older NSAIDs. An alternative result of increased overall NSAID prescription volume – where the number of selective COX-2 inhibitor prescriptions has increased by a greater amount than the associated reduction in the number of older NSAID prescriptions – might indicate that selective COX-2 inhibitors were being prescribed to certain population groups who would not previously have been prescribed an older NSAID.

O'Brien [6] has used the term 'leakage' to refer to the situation where once an intervention is provided for a specific indication and population group (for whom there is evidence of cost-effectiveness) it can 'leak' to other groups for whom it was not originally intended (and for whom it may also be less cost-effective). An example of leakage was given by Lopert [7], who pointed out that ACE inhibitors are more cost-effective for cardiac failure than for hypertension (they provide no clear benefit over beta blockers, but are considerably more expensive). Similarly, a retrospective examination of the appropriateness of proton pump inhibitor (PPI) prescribing found that in 49.8% of the cases examined patients who were prescribed a PPI did not meet the Australian government Pharmaceutical Benefits Scheme (PBS) prescribing criteria, criteria which was drawn up to restrict the use of PPIs on the grounds of cost-effectiveness [8]. More recently, NICE has recommended that herceptin be an option for women with advanced stage breast cancer [9], but women with early stage breast cancer are now also receiving the drug, even though herceptin is yet to be licensed for use, or evaluated in terms of cost-effectiveness, in this population group [10].

As well as recommending that selective COX-2 inhibitors only be an option for patients with arthritis who are at high risk of an adverse GI event, NICE also estimated that switching high-risk arthritis patients to selective COX-2 inhibitors would lead to an annual incremental expenditure of approximately £25 million to the National Health Service (NHS) [5]. This NICE guidance is consistent with other studies which have estimated that, compared to older NSAIDs, selective COX-2 inhibitors have a higher incremental cost, and that their provision is only cost-effective for groups at high risk of a GI event [11,12]. In this paper, we compare actual increases in overall NSAID expenditure to the £25 million per annum predicted by NICE. Evidence of expenditure increases beyond those that were predicted may also suggest that the provision of selective COX-2 inhibitors had leaked to population groups for whom its provision was not originally intended.

### Specific approach

In this paper we use data on the number of prescriptions, and their associated expenditure, as calculated by the Prescription Pricing Authority (PPA) (the PPA is responsible for processing all NHS prescriptions that are dispensed by any community pharmacy or dispensing doctor in England). Data for two selective COX-2 inhibitors (celecoxib and rofecoxib) and three older NSAIDs (diclofenac, ibuprofen, and naproxen) were collated. These two particular selective COX-2 inhibitors were chosen as they accounted for 78% of all selective COX-2 inhibitor prescription items in 2004 [1]. Similarly, diclofenac, ibuprofen, and naproxen were chosen as in 1998 (prior to the use of celecoxib and rofecoxib in England) they accounted for 77% of all NSAID prescription items [13]. In addition, diclofenac and ibuprofen were the comparators used in the largest randomised controlled trial (N = 8059) designed to assess the efficacy of celecoxib [14], and naproxen was the comparator used in the largest randomised controlled trial (N = 8076) designed to assess the efficacy of rofecoxib [15]. Data from both these clinical trials were submitted to the United States (US) Federal Drug Administration (FDA) for the purposes of licensing celecoxib and rofecoxib, respectively.

## Methods

### NSAID prescription volume

PPA data, for England, on the number of prescriptions for each individual NSAID were collated for quarterly (3 month) periods from January 1^st ^1999 (prior to the use of celecoxib and rofecoxib) to September 30^th ^2004: 23 quarters in total. Total quarterly prescription volumes for i) the two selective COX-2 inhibitors (celecoxib and rofecoxib), and ii) the three older NSAIDs (diclofenac, ibuprofen, and naproxen) were calculated for each of the 23 quarters. Bi-variate (Pearson) correlations were undertaken to compare total prescriptions volumes for the two selective COX-2 inhibitors to total prescription volumes for the three older NSAIDs over the 23 quarter period. Changes in the absolute prescription volume for both these groups were also calculated by comparing prescription volumes in the first quarter (ending March 1999) to those in the last quarter (ending September 2004).

### NSAID expenditure

PPA total prescription expenditure data, for England, was collated for the two selective COX-2 inhibitors (celecoxib and rofecoxib) and three older NSAIDs (diclofenac, ibuprofen, and naproxen) for the 23 quarters between January 1999 and September 2004. Similarly, the total increase in expenditure for the five NSAIDs, between the first and last quarter, was calculated. To account for the fact that, over time, higher NSAID prescription costs could have accounted for some of the increase in NSAID expenditure we also compared the average prescription cost for diclofenac, ibuprofen and naproxen in the last quarter to that in the first quarter. Average prescription costs, for each NSAID, were estimated by dividing the actual total prescription expenditure for the particular NSAID (in a particular time period) by the actual number of prescriptions made for the same NSAID (in the same time period).

Using the following methods, we also estimated the approximate increase in NSAID expenditure that arose due to i) substitution, and ii) increased NSAID prescription volume. To estimate the former, the reduction in the number of prescriptions for the three older NSAIDs (between the first and last quarter) was identified, and we assumed that the total number of selective COX-2 inhibitor prescriptions in the final quarter was equivalent to this reduction (i.e. that the total number of prescriptions for the five NSAIDs had remained constant between the first and last quarter), and that an equivalent number of prescriptions for celecoxib and rofecoxib were made. These quarterly prescription volumes for celecoxib and rofecoxib were then multiplied by their respective average prescription costs (for the last quarter), and summed together, in order to estimate what the total expenditure on the two selective COX-2 inhibitors (in the last quarter) would have been if the total number of prescriptions for the five NSAIDs had remained constant. This estimate of the expenditure on the two selective COX-2 inhibitors (based on constant NAID volume) was then added to the actual expenditure on diclofenac, ibuprofen and naproxen (in the last quarter) in order to estimate what the total expenditure on these the five NSAIDs would have been (in the last quarter), if the total prescription volume for these five NSAIDs had remained constant. Thus, the approximate increase in NSAID expenditure that arose due to substitution was then calculated by deducting the actual expenditure on the five NSAIDs in the first quarter, from the estimated expenditure on the five NSAIDs in the last quarter, based on a constant volume of prescriptions for these five NSAIDs.

The approximate increase in NSAID expenditure that arose due to increased NSAID prescription volume was estimated by deducting the previous (substitution) estimate of what the total expenditure on the five NSAIDs would have been in the final quarter (based on a constant nsaid volume) from the actual total expenditure on the five NSAIDs (in the final quarter). The increase in NSAID expenditure was thereby divided into that which was estimated to be accounted for by substitution (falling total diclofenac, ibuprofen, and naproxen volume), and that which could not (i.e. that which was a result of increasing overall NSAID volume). Finally, we compared these estimates to the NICE estimate that switching high-risk arthritis patients to selective COX-2 inhibitors would lead to an annual incremental NSAID expenditure of approximately £25 million.

## Results

### NSAID prescription volume

**Figure 1:**
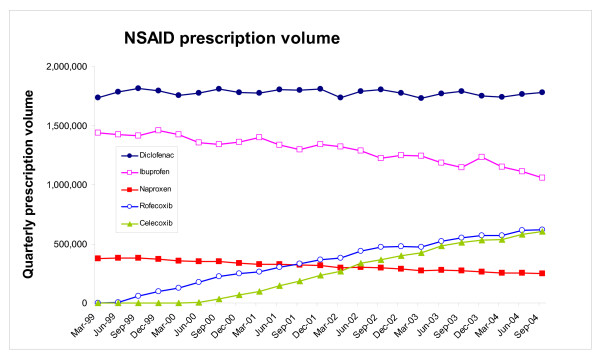
Individual NSAID quarterly NHS prescription volume England for the five NSAIDs (celecoxib, rofecoxib, diclofenac, ibuprofen and naproxen) between January 1999 and September 2004.

**Figure 2:**
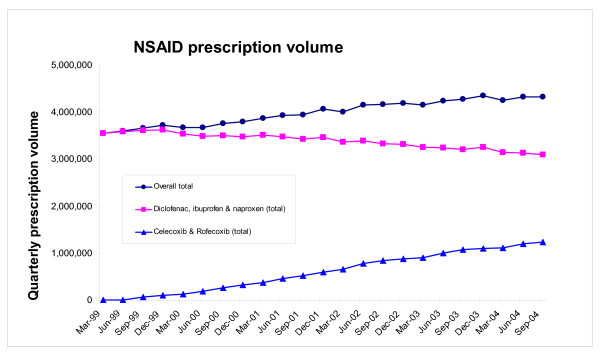
Total NSAID quarterly NHS prescription volume for England for i) celecoxib and rofecoxib, ii) diclofenac, ibuprofen, and naproxen, and iii) all five NSAIDs between January 1999 and September 2004.

By the last quarter (ending September 2004) prescription volumes for celecoxib and rofecoxib had reached levels of 606,409 and 620,790 items per quarter respectively (Figure 1). Figure 1 also shows that between the first quarter (ending March 1999) and the last quarter the number of prescriptions for ibuprofen and naproxen fell, but that the number of prescriptions for diclofenac remained relatively stable. Indeed between the first and last quarter total prescription volume for the two selective COX-2 inhibitors (celecoxib and rofecoxib) increased to 1.23 million per quarter, whilst prescription volumes for the three older NSAIDs (diclofenac, ibuprofen and naproxen) fell from 3.55 million to 3.09 million (Figure 2).

Total prescription volumes for the two selective COX-2 inhibitors, and three older NSAIDs were highly negatively correlated (r = -0.97, p < 0.001). Despite this, prescription volumes for three older NSAIDs fell by only 460,084 items per quarter between the first and last quarter, whereas the total number of prescriptions for two selective COX-2 inhibitors grew to 1,227,199 per quarter over the same period (Figure 2). As a consequence the cumulative number of prescriptions for the five NSAIDs grew from 3,550,249 in the first quarter to 4,317,364 in the last quarter – an increase of over 0.76 million items per quarter.

### NSAID prescription expenditure

**Figure 3:**
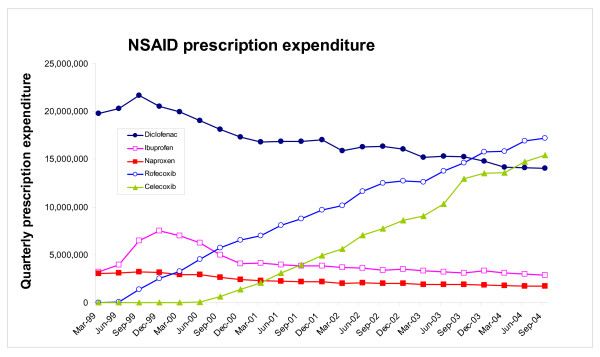
Individual NSAID quarterly NHS expenditure for England for the five NSAIDs (celecoxib, rofecoxib, diclofenac, ibuprofen and naproxen) between January 1999 and September 2004.

**Figure 4:**
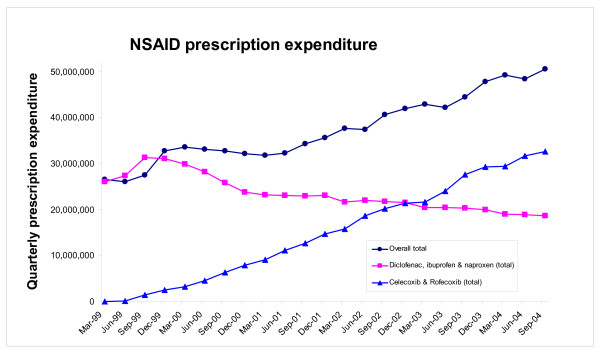
Total NSAID quarterly NHS expenditure for England for i) celecoxib and rofecoxib, ii) diclofenac, ibuprofen, and naproxen, and iii) all five NSAIDs between January 1999 and September 2004.

By the end of September 2004, expenditure on celecoxib and rofecoxib amounted to £15,430,892 per quarter and £17,221,195 per quarter, respectively, (Figure 3). Conversely, total expenditure on the three older NSAIDs fell from £26,583,346 in the first period to £18,671,535 in the final quarter (Figure 4). As such, over the 23 quarter period, overall expenditure on the five NSAIDs increased from £26,061,182 per quarter to £51,323,622 per quarter (Figure 4), an increase of £25.3 million per quarter. In the first quarter the average prescription costs for diclofenac, ibuprofen and naproxen were £11.53, £2.33 and £8.11, respectively, compared to £7.89, £2.71 and £7.02, respectively, in the last quarter. As these average prescription costs have generally fallen the increase in total NSAID expenditure can not be accounted for by an increase in average prescription costs (though the possibility that lower costs led to a higher volume than might otherwise have been the case can not be discounted). The average prescription costs for celecoxib and rofecoxib were £17.65 and £22.47 when they were first prescribed (in the quarters ending June 2000, and June 1999, respectively), compared to £25.45 and £27.74 in the final quarter.

To estimate the approximate increase in total NSAID expenditure that arose due to substitution we assumed that 230,042 prescriptions for both celecoxib and rofecoxib would have been made in the last quarter (prescription volume for diclofenac, ibuprofen and naproxen fell by 460,084 per quarter between the first and last quarter). By multiplying these volumes by the average prescription costs of £25.45 and £27.74, the total expenditure on celecoxib and rofecoxib would have been estimated to be £5,853,728 and £6,381,543, respectively (a combined total expenditure of £12,235,271 for the two selective COX-2 inhibitors). By adding this combined total expenditure to the actual expenditure on the three older NSAIDs in the final quarter (£18,671,535) we estimated that the expenditure on the five NSAIDs would have been £30,906,806 (if NSAID volume had remained constant between the first and last quarter). The actual expenditure on the five NSAIDs was £26,061,182 in the first quarter, thus we estimate that the approximate increase in NSAID expenditure that arose due to substitution was £4,845,625 per quarter (equivalent to £19,382,498 per annum).

To estimate of the approximate increase in NSAID expenditure that arose due to increased NSAID prescription volume, between the first and last quarter, we deducted the value of £30,906,806 (the estimated expenditure for the five NSAIDs based on constant volume) from the actual expenditure on the five NSAIDs in the last quarter (£51,323,622). Thus we estimate that the actual expenditure on the five NSAIDs was £20,416,816 per quarter higher than we estimate the expenditure on the five NSAIDs would have been if the last quarter total prescription volume for the five NSAIDs had been equivalent to that in the first quarter, and that increased volume accounted for 81% of the increase in NSAID expenditure. This largely arose because, based on a constant volume, total expenditure on the two selective COX-2 inhibitors would have been estimated to be £12,235,271 in the last quarter, whereas it actually amounted to £32,652,087 in the last quarter.

## Discussion

It has been shown that the introduction of two newer NSAIDs (celecoxib and rofecoxib) was associated with a fall in total prescription volume for three older NSAIDs (diclofenac, ibuprofen, and naproxen). However, for the five NSAIDs overall there were 0.76 million more prescriptions in the last quarter, compared to the first, and expenditure rose by £25.3 million per quarter (equivalent to an increase of over £100 million per annum).

Had the volume of prescriptions for the five NSAIDs remained constant over the 23 quarter period we estimate that due to substitution (from cheaper NSAIDs to more expensive selective COX-2 inhibitors) expenditure would have been £30.9 million in the last quarter, compared to £26.1 million in the first quarter (the difference of £4.85 million per quarter is equivalent to an annual increase of approximately £19.4 million). NICE estimated that switching high-risk arthritis patients to selective COX-2 inhibitors would increase NSAID expenditure by £25 million per annum. This figure is comparable to the increase in NSAID expenditure that we estimated was due to substitution (£19.4 million). The higher increase estimate by NICE may be partially explained by the fact that expenditure on the five NSAIDs in the quarter prior to the NICE report (quarter ending June 2001) was £23.1 million, compared to £26.1 million in the first quarter of our data set, and by the fact that our expenditure estimates were based on only two selective COX-2 inhibitors, compared to the four used by NICE [5]. Either way, the NICE estimate that switching patients to selective COX-2 inhibitors would increase NSAID expenditure by £25 million per annum is substantially below the actual increase in NSAID expenditure, which we estimated to be equivalent to over £100 million (for the five NSAIDs, between January 1999 and September 2004). Indeed we estimated that 81% of the increase in NSAID expenditure was due to increased NSAID volume.

### Explanations

In randomised trials the estimated number of adverse GI events for people taking the NSAIDs of naproxen or ibuprofen has been estimated to be between two and four times higher than the number for people taking rofecoxib [15-18]. Similarly, people taking diclofenac or ibuprofen were estimated to suffer between two and three times the number of adverse GI events as those taking celecoxib [14]. Consequently, it is not surprising that the release of celecoxib and rofecoxib coincided with a reduction in the number of prescriptions for naproxen and ibuprofen. We can not readily explain why prescriptions levels for diclofenac remained largely unchanged over the six year period, though the fact that the average prescription cost of diclofenac fell by 32% during this time may be a contributory factor. As overall volume for the five NSAIDs has increased by 0.76 million items per quarter this may however suggest that both celecoxib and rofecoxib could have leaked to population groups who would not previously have received a NSAID. People may not have been prescribed NSAIDs previously because, even though they were in pain, the potential benefits were judged not to outweigh the risk of an adverse GI event – a situation which presumably changed with the arrival of celecoxib and rofecoxib.

### Comparisons with other studies

Our results are in line with those from two recent studies. Joshua et al. [19] found that use of selective COX-2 inhibitors by rheumatology patients increased from 18% (3 months after their release) to 57% (16 months after their release). During the same period prescription rates for other NSAIDs fell from 43% to 20%. Moreover, Joshua et al. [19] found that prescribing patterns for selective COX-2 inhibitors were largely unrelated to the patient's estimated risk of an adverse GI event. Similarly, Dai et al. [20] found that 35% of patients at the lowest risk for adverse events from NSAIDs received a COX-2 inhibitor in 2002, compared to just 12% in 1999. In addition, when discussing the use of selective COX-2 inhibitors, the US Food and Drug Administration (FDA) concluded that they had been "...prescribed for indications and patients far beyond their original intent" [21].

### Study weaknesses

One of the main weaknesses of this study is that we have looked at prescription items, and not the number of patients taking each individual NSAID. Moreover, the number of prescription items does not take account of the dosage or quantity of the drug prescribed [22]. Thus though we know that the number of prescriptions for the five NSAIDs has increased by 0.76 million items per quarter we can not be sure that more patients have been prescribed a NSAID (it may be that the same patients are receiving more prescriptions than previously). Moreover this increased volume could be due to confounding factors that have not been controlled for in this 6 year study period. For example, it has been estimated by Harkness et al. [23] that over the past 40 years the prevalence of musculoskeletal pain has increased by between 2 and 4 fold, though it was acknowledged that this could be due to increased awareness and reporting. Either way, this means that some other factor (e.g. increased prevalence), rather than the release of celecoxib and rofecoxib, could partially account for the increased volume of NSAID prescriptions. That said, weight is added to the argument that there is a substitution effect between selective COX-2 inhibitors and older NSAIDs, by the fact that prescription volumes for diclofenac, ibuprofen and naproxen were increasing prior to the fall that occurred after the release of celecoxib and rofecoxib (in total, there were 14.42 million prescriptions for these three older NSAIDs in 1999 [24], compared to 14.15 million in 1998 [13]).

One of the five NSAIDs (ibuprofen) is available without prescription (often referred to as over-the-counter (OTC)), but we did not monitor how OTC sales of ibuprofen have changed since the release of celecoxib and rofecoxib. Were OTC sales of ibuprofen to have increased over the 6 year period then this, rather than the release of celecoxib and rofecoxib, could account for some of the reduction in ibuprofen prescriptions. This in turn would however mean that the level of NSAID volume would have increased by an even greater amount than that estimated in this study.

Finally, readers should be aware that rofecoxib has recently been withdrawn from the market by its manufacturer Merck [25]. This was due to evidence of an increased risk of serious coronary heart disease when taking rofecoxib, particularly at high dosages and for prolonged periods [26,27]. Some studies (e.g. [28]), but not others (e.g. [29]), have also found that the use of celecoxib increases a person's risk of coronary heart disease. However, after considering all available evidence, the US FDA decided to not to withdraw celecoxib and other selective COX-2 inhibitors from the market [30]. Given that rofecoxib was withdrawn after the period for which data were collected within this study we do not believe that this impinges on the results of this study. Indeed celecoxib and rofecoxib were the main two selective COX-2 inhibitors in England in 2004, accounting for 74% of NHS expenditure on selective COX-2 inhibitors [1]. Moreover, it was the evidence of increased risk of coronary heart disease in a group for which the rofecoxib was not originally intended (bowel cancer patients) that led to its withdrawal [26].

## Conclusion

It has been shown that the introduction of celecoxib and rofecoxib coincided with a reduction in the total number of prescriptions for diclofenac, ibuprofen and naproxen. However, between January 1999 and September 2004, overall prescription volume for the five NSAIDs increased by 0.76 million items, and expenditure was estimated to have increased by £20.4 million per quarter more than would have been expected if overall NSAID volume had remained constant. This suggests that celecoxib and rofecoxib may have leaked to population groups who would not previously have received a NSAID.

## Competing interests

The author(s) declare that they have no competing interests.

## Authors' contributions

GRB had the original idea for the study, undertook the statistical analyses and drafted the paper. Both AJA and DKW made revisions to the paper. All authors have given final approval for this version of the paper to be published.
